# Disturbed Relaxin Signaling Pathway and Testicular Dysfunction in Mouse Offspring upon Maternal Exposure to Simazine

**DOI:** 10.1371/journal.pone.0044856

**Published:** 2012-09-12

**Authors:** Ho-Oak Park, Jeehyeon Bae

**Affiliations:** College of Pharmacy, Chung-Ang University, Seoul, Korea; National Cancer Institute, United States of America

## Abstract

Simazine is a triazine herbicide that is being widely applied worldwide and commonly detected in surface and groundwater. Despite its popular use in controlling weeds and algae, very limited information is available regarding its toxicity. In the present study, pregnant mice were orally exposed to low doses (0, 5, 50, or 500 µg/kg body weight per day) of simazine during gestation and lactation, during which no overt maternal toxic response was detected, and their offspring was assessed. Simazine-exposed male offspring showed decreased body, testicular, and epididymis weight, increased testicular apoptosis, and decreased sperm concentrations. Differentially-expressed genes in the testes of male offspring exposed to simazine were identified by DNA microarray, revealing 775 upregulated and 791 downregulated genes; among these, the relaxin-family peptide receptor 1 (Rxfp1), which is the receptor for relaxin hormone, was significantly downregulated. In addition, the expression of target genes in the relaxin pathway, including nitric oxide synthase 2 (Nos2) and Nos3, was significantly decreased in simazine-exposed F1 testes. Moreover, simazine inhibited NO release, and knockdown of Rxfp1 blocked the inhibitory action of simazine on NO production in testicular Leydig cells. Therefore, the present study provides a better understanding of the toxicities associated with the widely used herbicide simazine at environmentally relevant doses by demonstrating that maternal exposure interferes with the pleotropic relaxin-NO signaling pathway, impairing normal development and reproductive activity of male offspring.

## Introduction

Simazine (6-chloro-*N,N′*-diethyl-1,3,5-triazine-2,4-diamine) is an herbicide of the triazine family, which also includes atrazine and propazine, and has been applied worldwide in both agricultural and nonagricultural uses, including treatment on a diversity of deep-rooted crops to control broad-leaved and grassy weeds and on algae in farm ponds for pre-emergence purposes [1]. In the United States, an estimated 5 to 7 million pounds of simazine are applied to agricultural crops each year, and an additional 1.2 million pounds are applied for nonagricultural uses [1]. Exposure routes of simazine include contaminated drinking water, dermal contact, and inhalation from occupational exposure [2]. Simazine is one of most commonly detected pesticides in surface and ground water due to herbicide runoff [3]. Simazine has been detected in the urine of pregnant women at a median concentration of 1 µg/L [4].

The toxicity of structurally related chlorinated triazines is considered low, and the U.S. Environmental Protection Agency (EPA) has performed a cumulative risk assessment of the chlorinated triazine-class chemicals and their degradation products and concluded that the cumulative risks associated with the compounds are below the EPA’s level of concern [1]. However, numerous recent *in vivo* and *in vitro* studies have reported an array of toxic responses to atrazine, a close homologue of simazine, affecting neuroendocrine systems, antioxidant mechanisms, behavior, and mammary gland development [5]–[9].

In 2009, simazine was included in the final list of chemicals tested in an endocrine disruptor screening program (EDSP) by the U.S. EPA due to its characteristics of multiple exposure pathways and high production volume. Endocrine disruptors (EDs) are exogenous agents that interferes with the synthesis, secretion, transport, binding, action, or elimination of natural hormones that are responsible for the maintenance of homeostasis, reproduction, development, and/or behavior [10]. To date, toxicological data for simazine are scarce; in particular, mammalian multigenerational studies after *in vivo* exposures of low doses of simazine during fetal and neonatal periods have not been reported. The “no observed adverse effect levels” (NOAELs) for acute and chronic dietary exposure of simazine in all populations are 30 mg/kg/day and 1.8 mg/kg/day, respectively, and US EPA’s Office of Water established a Maximum Contaminant Level (MCL) for simazine in finished drinking water of 4.0 parts per billion (ppb) [1]. Based on these facts, we selected very low simazine doses at 5, 50, and 500 µg/kg body weight per day by gavage in the present study. The development of the reproductive system is under tight hormonal regulation, and fetal and neonatal stages are the most vulnerable periods for proper development. Therefore, we assessed the risks of maternal exposure to low concentrations of simazine during these developmental periods and found that simazine exerts testicular toxic responses in male offspring involving the relaxin-family peptide receptor 1 (Rxfp1)-mediated nitric oxide (NO) signaling pathway.

## Materials and Methods

### Chemicals

Simazine (CAS No. 122-34-9; 99.9% pure) was purchased from Sigma-Aldrich Laborchemikalien GmbH (Wunstorfer Str. 40, Seelze, Germany), and the other chemicals used in the experiments were purchased from Sigma (St. Louis, MO, USA) unless otherwise indicated.

### Animals and Simazine Exposures

**Figure 1:**
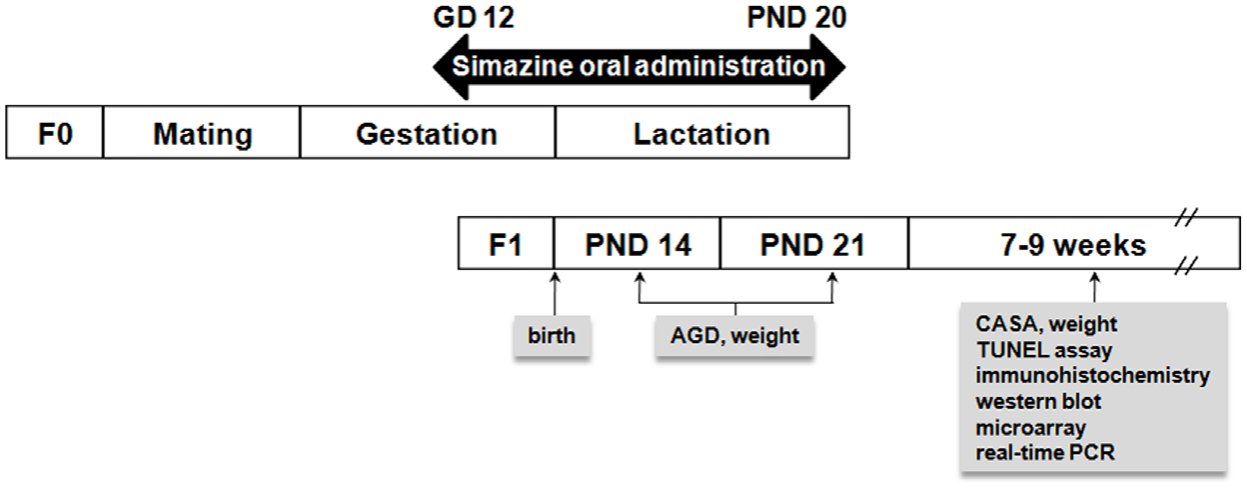
Schematic of the *in vivo* animal experiment. Dams (F0) were mated, and pregnant mice were exposed to different daily doses of simazine from gestational day (GD) 12 to postnatal day (PND) 20. An assessment of body and organ weights, anogenital distance (AGD), computer-assisted sperm analysis (CASA), immunohistochemistry, western blot, microarray and real-time PCR of their male offspring (F1) was performed at the indicated time points.

Eleven-week-old virgin C57BL/6 female mice and eighteen-week-old DBA/2 male mice were obtained from SLC, Inc. (Tokyo, Japan). The acclimatization period was 3 days, and the mice were mated to obtain F1 offspring. The animal room in which all mice were housed was maintained at a humidity of 30–40% and a temperature of 22±1°C. The lighting in the room was on a 12-h light/dark cycle. All animals were given water *ad libitum* and AIH-76A rodent feed (Research Diets, New Brunswick, NJ, USA). The animals were treated humanely and handled so as to minimize their suffering, according to the experimental protocol approved by the CHA University Institutional Animal Care and Use Committee. Female mice were given simazine (0, 5, 50 or 500 µg/kg body weight per day) by oral administration in 0.1 ml corn oil by daily gavage from gestation day (GD) 12 to postnatal day (PND) 20 (Fig. 1).

### Animal Assessment

Mice at PNDs 14 and 21 and 7–9-week-old male offspring (F1) and their dams (F0) were euthanized by cervical dislocation; the bodies and organs were weighed. Collected tissue samples were either submerged in RNase-free water pretreated with 0.1% (v/v) diethyl pyrocarbonate (DEPC) and then snap-frozen in liquid nitrogen gas or stored at 4°C in 10% formaldehyde (Sigma, Steinheim, Germany). The anogenital distance (AGD) of the offspring was measured on PNDs 14 and 21. AGD was measured from the center of the anus to the junction of the smooth perineal skin with the rugated skin of the scrotum in male mice.

### Computer Assisted Sperm Analysis (CASA)

The cauda distal epididymides were punctured with a 25-gauge needle in a 60 mm organ culture dish (Orange Scientific Inc, Belgium) containing 1.0 ml of pre-warmed Dulbecco’s modified Eagle medium with Ham’s F-12 nutrient mixture (DMEM/F-12; Welgene, Seoul, Korea) supplemented with 10% heat-inactivated fetal bovine serum (FBS; Welgene). Sperm concentration and motion analyses were conducted within 30 min after puncture. Quantitative parameters of sperm motility were assayed in randomly chosen fields of the hemocytometer and measured by CASA using the integrated visual optical system (IVOS) motility analyzer (Hamilton-Thorne Research Inc., Beverly, MA, USA), software version 10.7. Quantitative parameters of sperm motility evaluated in this study were the standard mouse parameters as recommended by the manufacturer, which were sperm concentration, % motile sperm, smoothed path velocity (VAP), straight line velocity (VSL), track velocity (VCL), amplitude of lateral head displacement (ALH), beat cross frequency (BCF), straightness (STR), linearity (LIN), and % elongation.

### Immunohistochemistry

Testes from 8-week-old F1 male mice were immersion-fixed in 4% neutral buffered formalin and then stored at 4°C until analysis. Paraffin-embedded testes were sectioned to a thickness of 5 µm using a FINESSEE microtome (Thermo Scientific, Rockford, IL, USA) and deparaffinized. The sections were rehydrated, immersed in an antigen-retrieval solution (0.01 M sodium citrate and 0.05% Tween 20; pH 6.0), and microwaved for 10 min (100°C at 600 W). Endogenous peroxidase was blocked by immersing the sections in a 3% hydrogen peroxidase solution (Duksan, Ansan, Korea) for 10 min, followed by rinsing in phosphate-buffered saline (PBS). Nonspecific staining was blocked with 2.5% normal horse serum (Vector Laboratories, Burlingame, CA, USA) for 1 h at room temperature. The sections were then incubated with polyclonal anti-human Rxfp1 (1∶100) (Santa Cruz Biotechnologies, Santa Cruz, CA, USA) in the antibody diluent (Dako, Carpinteria, CA, USA) for 45 h at 4°C. For the incubation with secondary antibody, the Universal ImmPRESS™ REAGENT (Vector Laboratories) was used as directed by the manufacturer. All sections were counterstained with Mayer’s hematoxylin solution for 5 min. The slides were then dehydrated and mounted for microscopic observation.

### Terminal Deoxynucleotidyl Transferase dUTP Nick End Labeling (TUNEL) Assay

DNA fragmentation in the testes of 8-week-old mice was assessed *in situ* by TUNEL (Roche, Mannheim, Germany) as described by the manufacturer, with minor modifications. Paraffin sections of 5-µm thickness were treated with proteinase K (20 µg/mL) for 15 min at room temperature after blocking of the endogenous peroxidase activity with 3% hydrogen peroxide for 10 min. After washing in 0.01 M PBS, the slides were incubated in TUNEL Mix comprising 0.01 U/µL terminal transferase, 1 mM CoCl_2_, 0.4 mM digoxigenin deoxyuridine triphosphate, and reaction buffer (200 mM KCl, 25 mM Tris-HCl, 0.25 mg/mL bovine serum albumin, pH 6.6) for 1 h at 37°C. The reaction was terminated by washing in 0.2% saline-sodium citrate (30 mM NaCl, 3 mM sodium citrate, pH 7.4). The sections were then treated with anti-digoxigenin peroxidase-conjugated sheep polyclonal antibody (1∶500) for 30 min at 37°C. Slides were washed in PBS, and color was developed using 3,3′-diaminobenzidine (DAB). The slides were then counterstained with hematoxylin. Cells exhibiting brown staining were considered to be positive for apoptosis-associated DNA fragmentation. Negative controls were processed in a similar manner, with the omission of terminal transferase in the reaction mixture.

### RNA Preparation and Microarrays

Total RNA was isolated from ten testes (8-week-old), one from each of the five different control mice and the five mice maternally exposed to simazine at 500 µg/kg per day using TRIzol reagent (Invitrogen, Carlsbad, CA, USA) according to the manufacturer’s protocol. For quality control, RNA purity and integrity were evaluated by denaturing gel electrophoresis, OD 260/280 ratio, and analysis on an Agilent Bioanalyzer 2100 which is a microfluidics-based platform for sizing, quantification and quality control of DNA, RNA, proteins, and cells. (Agilent Technologies, Palo Alto, CA, USA). Equal amounts of RNA from each individual testis in each group were pooled. The array was performed using the Roche NimbleGen mouse whole-genome 12-plex array (Roche NimbleGen, Inc., Madison, WI, USA) covered by a NimbleGen H12 mixer (Roche NimbleGen, Inc.).

### Cell Culture

Rat testicular Leydig cell line LC540 (Korean Cell Line Bank, Seoul, Korea) was cultured in minimum essential medium (PAA Laboratories, Etobicoke, Canada). Media contained 10% fetal bovine serum (FBS) (PAA Laboratories) and 1% penicillin-streptomycin (Welgene, Seoul, Korea).

### Quantitative Real-time PCR (qRT-PCR)

Total RNAs from mouse testes and LC540 cells were isolated by the same isolation method using TRIzol reagent, and the concentration and quality of RNA were determined with an ND-1000 spectrophotometer (NanoDrop, Waltham, MA, USA). Reverse-transcription to cDNA was performed using the SuperScriptIII first-strand synthesis kit (Invitrogen), following the manufacturer’s instructions. All cDNAs used in real-time PCR were normalized with β-actin. Quantitative real-time PCRs were performed using an iQ™ SYBR Green Supermix (Bio-Rad Laboratories, Benicia, CA, USA). Gene expression was quantified by the delta-delta-CT method, and real-time PCRs were performed in a CFX-96™ thermal cycler and detection system (Bio-Rad Laboratories). The nucleotide sequences of the primers (Bioneer, Daejon, Korea) used are as listed in Table 1.

**Table 1:** Oligonucleotide Sequences of Primers Used.

Gene	Sequence	
β-actin	Mouse	F : GGGTCAGAAGGACTCCTATG
		R : CACGGTTGGCCTTAGGGTTCA
	Rat	F : AACTTTGGCATCGTGGAAGG
		R : AAGTCACAGGAGACAACC
Bcl-2(b-cell leukemia/lymphoma 2)	Mouse	F : CGTCGTGACTTCGCAGAGAT
		R : CTCCACACACATGACCCCAC
Bcl-xl(bcl2-like 1)	Mouse	F : GACAAGGAGATGCAGGTATTGG
		R : TCCCGTAGAGATCCACAAAAGT
Bcl-w(bcl2-like 2)	Mouse	F : GCTGCTGGAGACGAGTTTGA
		R : ACTCTCAGCACACAGGGCAG
Bfl-1(b cell leukemia/lymphoma 2 related protein A1a)	Mouse	F : TGGCATCATTAACTGGGGAAGG
		R : AGCACATACATCCAGGGCAAT
Mcl-1(myeloid cell leukemia sequence 1)	Mouse	F : GACGACCTATACCGCCAGTC
		R : TCGCCTTCGTTTTTAATGTCCA
Bax(bcl2-associated X protein)	Mouse	F : TGAAGACAGGGGCCTTTTTG
		R : AATTCGCCGGAGACACTCG
Bak(bcl2-antagonist/killer )	Mouse	F : AGCAGGTTGCCCAGGACACA
		R : TCCGATGAGAGCAAGCTGCC
Bad(bcl2-associated agonist of cell death)	Mouse	F : TGAGCCGAGTGAGCAGGAAG
		R : TGAGCCGAGTGAGCAGGAAG
Bim(bcl2-like 11)	Mouse	F : CCCTGGCCCTTTTGCTACC
		R : ACTTGTCACAACTCATGGGTG
Rxfp1(relaxin family peptide receptor 1)	Mouse	F : TGTCCCCTCGGCTCCTTCCC
		R : CCTCATCAGCTCGGTTCCCGC
	Rat	F : ACAAGCTGCACGCCATGTCCA
		R : CATCCACGGCTGTGCGTGCTT
Nos2(nitric oxide synthase 2)	Mouse	F : GTTCTCAGCCCAACAATACAAGA
		R : GTGGACGGGTCGATGTCAC
	Rat	F : CGGCTGCCCGGAAAACCCAA
		R : ACATCCCGAGCCATGCGCAC
Nos3(nitric oxide synthase 3)	Mouse	F : GCAATCTTCGTTCAGCCATCA
		R : CCAGCCATGTTGGATACAGAG
Vegf(vascular endothelial growth factor)	Mouse	F : GCGAAGCTACTGCCGTCCGA
		R : GCGAAGCTACTGCCGTCCGA
Mmp9(matrix metalloproteinase 9)	Mouse	F : CTGGACAGCCAGACACTAAAG
		R : CTCGCGGCAAGTCTTCAGAG

### Western Blot Analysis

Tissue samples were homogenized using a PRO 200 homogenizer (PRO Scientific Inc., Oxford, CT, USA) in cold PRO-PREP™ solution (Intron, Seongnam, Korea). Equal amounts of total protein were subjected to sodium dodecyl sulfate-polyacrylamide gel electrophoresis (SDS-PAGE) and transferred to membranes. The equal loading of proteins was confirmed by probing the same membrane with a monoclonal antibody against β-actin (Santa Cruz Biotechnology) at 1∶1,000. Membranes were stripped and incubated overnight with polyclonal antibodies against Rxfp1 and Nos2 (Santa Cruz Biotechnology). After washing, the membrane blots were incubated at room temperature for 2 h with horseradish peroxidase-conjugated anti-rabbit IgG secondary antibody at a 1∶3,000 dilution (Santa Cruz Biotechnology) before visualization by enhanced chemiluminescence (AB Frontier, Seoul, Korea) and detection using a Fujifilm LAS3000 Intelligent Dark Box Imager (Fuji, New York, NY, USA). Relative expression (%) was calculated based on the protein densities of the immunoprecipitates, as determined with MultiGauge V 3.0 software (Fuji).

**Table 2:** Reproductive Performance of F0 Female Mice Exposed to Simazine.

Maternal doses of simazine (µg/kg/day)	0	5	50	500
**Number of pregnant mice**	8	8	8	9
Number of viable litters	8	8	8	9
Fecundity^a^	100	100	100	100
**Body weight**				
Pre-mating	21.58±0.39	20.74±0.39	20.88±0.39	20.74±0.37
PND1	27.11±0.83	27.67±0.83	26.41±0.83	25.79±0.83
PND7	27.98±0.76	27.01±0.76	28.18±0.76	27.74±0.76
PND14	28.83±0.76	27.59±0.81	29.30±0.76	28.49±0.76
PND21	28.57±1.12	27.16±1.04	28.84±1.04	28.77±1.12
Weight gain^b^	6.77±1.10	6.64±1.02	7.93±1.02	8.15±1.10
Number of alive pup (PND 0)	8.1±1.1	7.8±1.1	8.6±1.1	8.3±1.2
Number of stillborn pup (PND 0)	0.9±0.7	1.3±0.7	0.0±0.7	0.4±0.7
Litter size (PND 1)	8.1±1.1	7.8±1.1	8.6±1.1	8.3±1.2
Litter weight (PND1, g)	11.01±1.10	11.43±1.13	11.43±1.06	12.65±1.06
Average pup weight (PND 1, g)	1.37±0.05	1.29±0.05	1.36±0.05	1.35±0.05
Pup survival rate on PND 4^c^	83.0±11.9	78.6±11.9	98.9±11.9	75.5±11.2
Pup survival rate on PND14^d^	83.0±12.8	67.0±13.7	98.9±12.8	75.5±12.1
Percent males	49.6±5.2	53.3±6.2	58.6±4.9	50.1±5.2

All values are raw or LS means mean ± SE.

a (Number of pregnant females giving birth to live young/number of pregnant females)×100, %.

b Difference between pre-mating body weight and final body weight on PND 21.

c (Number of pup alive on PND 4/number of pups alive PND 1)×100, %.

d (Number of pups alive on PND 14/number of pups alive on PND 1)×100, %.

### RNA Interference

Endogenous Rxfp1 was silenced using the specific oligonucleotides (Invitrogen) of 5′-GGUCUCUACAACUGGACAA and 5′-UUGUCCAGUGTAGAGACC, while 5′-CCUACGCCACCAAUUUCGU and 5′-ACGAAAUUGGUGGCGUAGG were used as a control. The sense and antisense oligonucleotides were annealed in the presence of Annealing Buffer (Bioneer). LC540 cells (3×10^5^) were resuspended in Resuspension R Buffer (Digital Bio Technology, Seoul, Korea), electroporated with 200 nM of siRNA specific for Rxfp1 or scrambled using a MicroPorator MP-100 (Digital Bio Technology), and incubated on plates containing fresh media, and knockdown was assessed by western blotting approximately 48 h after transfection.

**Table 3:** Body Weight and Anogenital Distance (AGD) of F1 Male Mice Exposed to Simazine Maternally.

Maternal doses of simazine (µg/kg/day)	0	5	50	500
Male body weight (g)				
PND 14	6.34±0.13 (30/7)	5.89±0.14* (27/6)	6.55±0.12 (35/7)	5.99±0.13* (29/6)
PND 21	9.00±0.23 (23/7)	8.52±0.23 (22/6)	8.76±0.19 (33/6)	8.34±0.23* (24/6)
Male AGD (mm)				
PND 14	2.61±0.11 (30/7)	2.45±0.11 (27/6)	2.53±0.10 (35/7)	2.67±0.11 (29/6)
PND 21	3.83±0.11 (23/7)	3.73±0.11 (22/6)	3.64±0.09 (33/6)	3.73±0.11 (24/6)

All values are means ± SEM.

Significantly different from control; *p*<0.05*.

Parentheses are the number of animals/number of litters.

**Table 4:** Body and Organ Weights of Young Adult F1 Male Mice Exposed to Simazine.

Maternal doses of simazine (µg/kg/day)	0	5	50	500
Number of animals/number of litters	9/5	5/3	5/2	4/2
Body weight (g)	26.97±1.31	24.54±0.71	20.02±0.90	20.45±0.45
Absolute organ weights (mg)				
Paired testis	0.2214±0.0125	0.1553±0.0186*	0.1674±0.0033*	0.1406±0.0031*
Paired epididymis	0.0691±0.0030	0.0661±0.0078	0.0552±0.0060	0.0455±0.0017*
Paired Cowper’s gland	0.1178±0.0130	0.1060±0.0035	0.0875±0.0059	0.0779±0.0105*
Seminal vesicle	0.2264±0.0192	0.2061±0.0242	0.1247±0.0308*	0.1872±0.0127
Thymus gland	0.0687±0.0045	0.0509±0.0064	0.0273±0.0045*	0.0429±0.0034*
Adjusted organ weight (mg/g)^a^				
Paired testis	0.0081±0.0008	0.0063±0.0007	0.0084±0.0003	0.0069±0.0003
Paired epididymis	0.0026±0.0001	0.0027±0.0003	0.0028±0.0004	0.0022±0.0001*
Paired Cowper’s gland	0.0044±0.0004	0.0044±0.0002	0.0043±0.0001	0.0038±0.0005
Seminal vesicle	0.0171±0.0086	0.0083±0.0008	0.0061±0.0013	0.0092±0.0006
Thymus gland	0.0069±0.0044	0.0021±0.0003	0.0013±0.0001*	0.0021±0.0002

All values are means ± SEM.

a (organ weight/body weight at the time of necropsy).

Significantly different from control; *p* < 0.05*.

### Measurement of NO Production

Nitrite was measured using the Griess Reagent System (Promega, Madison, WI, USA) as directed by the manufacturer. Briefly, LC540 cells were plated and incubated, and the medium was collected at 36 h after simazine exposure and centrifuged. The supernatant was analyzed for nitrite concentration based on a nitrite standard reference curve.

### Statistical Analysis

Data analysis was performed using SAS version 8.0 (SAS Institute, Cary, NC, USA). All data were tested for normality by the Shapiro-Wilk test at the 5% level of significance. Non-normal data were log transformed and retested. Nonparametric one-way analysis of variance (ANOVA) using the NPAR1WAY procedure in SAS was performed for non-normal data. The Kruskal-Wallis test was used to compare between the control and test groups. Data passing the normality test were analyzed with a repeated-measures ANOVA using the PROC GLM procedure in SAS. Dunnett’s and Student’s *t*-tests were performed for comparison of the control and treatment groups. Statements of significance are based on *p* values of less than 0.05.

**Table 5:** Epididymal Sperm Concentration and Quality of Young Adult F1 Offspring Exposed to Simazine.

Maternal doses of simazine (µg/kg/day)	0	5	50	500
Number of animals/number of litters	5/3	5/3	5/2	4/2
Sperm concentration (M/ml)	37.10±5.12	27.63±3.56	16.01±3.83*	18.59±3.27*
Motile sperm (%)	46.80±10.25	42.80±8.99	44.50±8.69	52.0±7.73
Progressive (%)	11.60±3.05	11.90±3.48	13.40±2.99	9.11±1.99
VAP (µm/s)	64.58±6.45	55.91±7.13	73.14±14.26	62.47±7.64
VSL (µm/s)	50.31±6.14	44.34±5.23	58.98±12.52	49.92±6.51
VCL (µm/s)	108.75±9.25	94.33±10.32	113.43±17.20	108.01±10.15
ALH (µm)	5.04±0.98	3.63±0.60	4.83±0.89	3.73±0.71
BCF (Hz)	22.51±1.90	19.11±3.18	17.86±3.05	22.69±2.62
STR (%)	76.90±1.04	81.00±2.49	77.90±2.34	78.00±1.98
LIN (%)	47.40±2.25	49.90±3.30	51.70±3.40	46.89±2.34
Elongation (%)	92.60±1.86	93.10±2.34	84.30±4.08	88.33±2.02

All values are means ± SEM.

Significantly different from control; *p* < 0.05*.

VAP: Smoothed path velocity (microns/sec).

VSL: Straight line velocity (microns/sec).

VCL: Track velocity (microns/sec).

ALH: Amplitude of lateral head displacement (microns).

BCF: Beat cross frequency (hertz).

STR: Straightness (ration of VSL/VAP).

LIN: Linearity (ratio of VSL/VCL).

Elongation: head shape (ratio of minor to major axis of sperm head).

## Results

### Reproductive Performance of F0 Dams Exposed to simazine

Dams exposed to 5, 50, or 500 µg/kg/day of simazine showed neither general toxicity nor significant changes in reproductive performance compared with the corn oil-fed control group. These results suggest that the doses of simazine used in the present study are low enough to not elicit maternal toxicity (Table 2).

### Decreased Body Weight of Simazine-exposed F1 Neonates

Although the body weights of mothers exposed to simazine were not affected, the body weights of their male offspring exposed to 5 or 500 µg/kg/day simazine *in utero* and by lactation were significantly reduced, by approximately 7% on PND 14, and there was a significant decrease in body weight for the offspring exposed to 500 µg/kg/day simazine on PND 21 (Table 3). The AGD, a sensitive marker used to assess reproductive toxicity, of the male littermates exposed to simazine was not significantly different from that of the control mice (Table 3).

### Decreased Body and Organ Weights in Simazine-exposed Young Adult F1 Male Mice

Body weights continued to be reduced in young male offspring maternally exposed to 50 or 500 μ g/kg/day of simazine by approximately 25%, although this decrease was not statistically significant (Table 4). Regardless, the absolute testicular weight was significantly decreased for all doses of simazine exposure, and the weights of other endocrine and reproductive organs including epididymis, Cowper’s gland, seminal vesicle, and thymus gland were also decreased in the simazine group (Table 4). The general trend of decreased organ weight remained even after weight adjustment (Table 4).

### Decreased Sperm Concentration and Quality in Simazine-exposed Young Adult Male Offspring

The epididymal sperm of the young male offspring was collected and analyzed by CASA. The sperm concentrations of the F1 males exposed to 50 or 500 µg/kg/day of simazine were significantly reduced to approximately 50% of the control (Table 5). Other parameters that measure the quality of sperm were not critically altered by simazine exposure (Table 5).

### Increased Testicular Apoptosis in Simazine-exposed Young Adult Male Offspring

**Figure 2:**
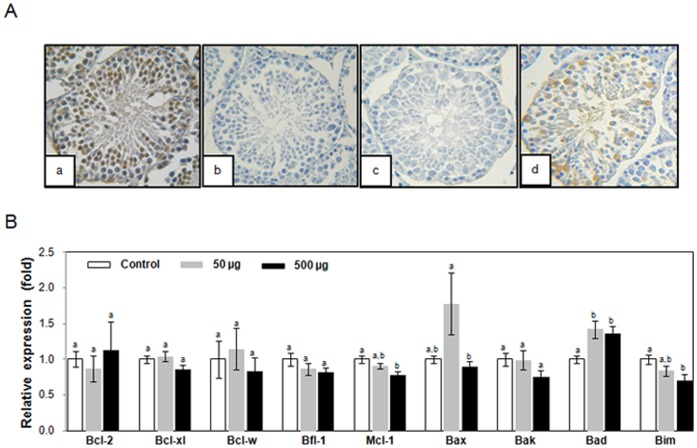
Increased testicular and ovarian apoptosis of mouse offspring exposed to simazine. (A) TUNEL-positive control section pretreated with DNase I (a), TUNEL-negative control section without terminal deoxynucleotidyl transferase treatment (b). The TUNEL assay was conducted on the cross-sections of 8-week-old mice testes (c and d) prepared from groups exposed to corn oil (c) or simazine (50 µg/kg) (d). (B) Real-time PCR analysis of Bcl-2 family members in the testes of control and simazine-exposed offspring was shown. The data were normalized to the expression level of β-actin and are presented as relative fold changes. The results are the mean ± SEM of six independent testicular analyses performed in triplicate for each group.

Therefore, to assess the effects of simazine on the testes of young adult male offspring, the testes were sectioned and cellular apoptosis was assessed. Strikingly, the number of TUNEL-positive apoptotic cells with brownish staining was prominently increased in simazine-exposed mouse testes (Fig. 2A), suggesting that maternal exposure to simazine induced testicular apoptosis of the offspring. Because Bcl-2 family proteins are central regulators of cellular apoptotic cascades in a diverse number of species [11], we next assessed if expression of any particular members of the Bcl-2 family were affected by simazine exposure. Although the mRNA expression of Bad, Bim, and Mcl-1 was moderately altered, generally the expression of majority of Bcl-2 family members did not show prominent changes in the simazine-exposed testes (Fig. 2B), suggesting that modulation of the expression of Bcl-2 family by simazine is unlikely the mechanism responsible for the apoptosis.

### Identification of Differentially Expressed Genes (DEGs) in Simazine-exposed F1 Testes

**Figure 3:**
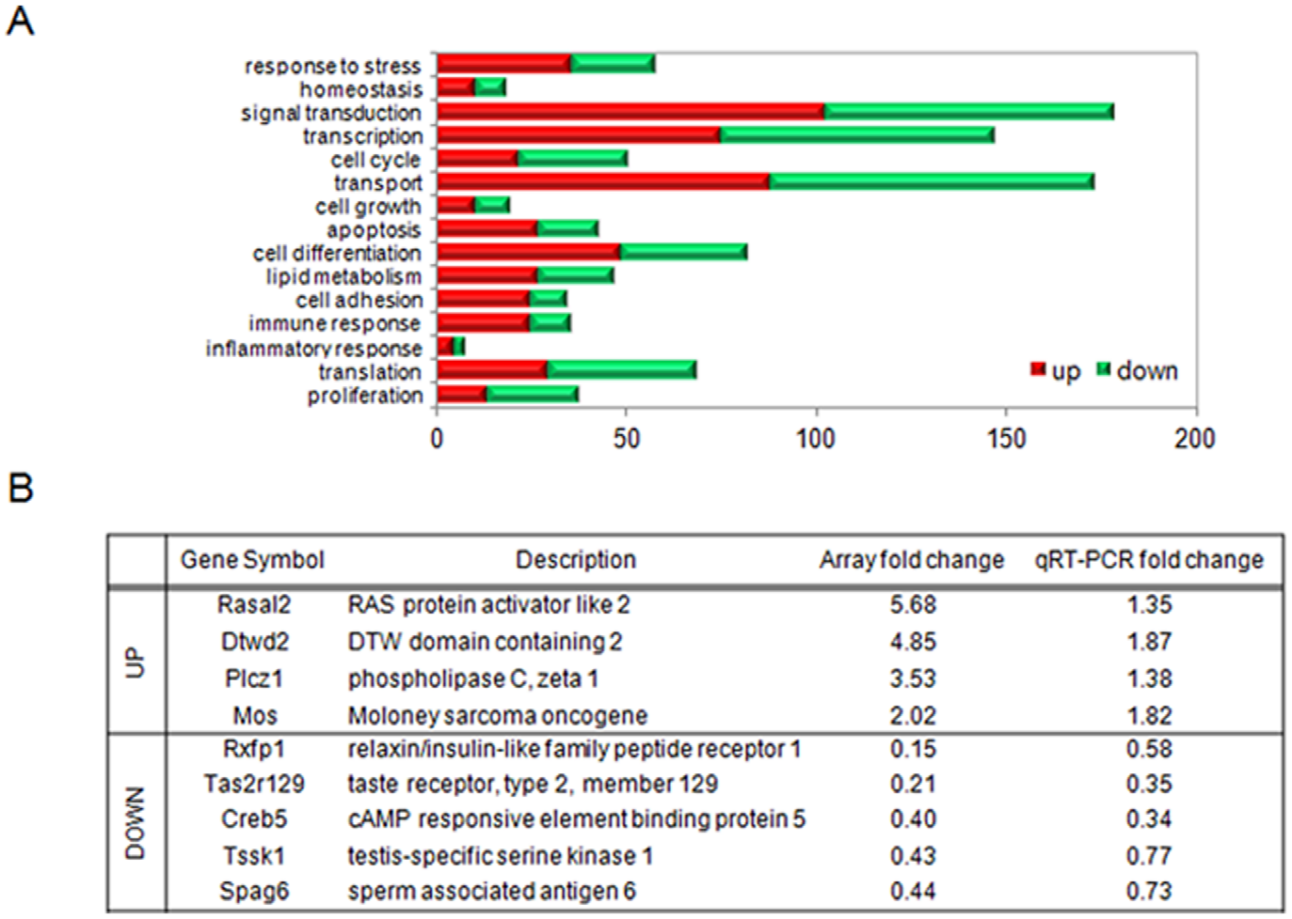
Summary and confirmation of microarray analysis. (A) Positively or negatively regulated DEGs in the testes of simazine (500 µg/kg)-exposed adult F1 mice compared with the control, classified by their roles in biological processes. (B) The expression levels of several of the differentially upregulated genes in the testes of F1 offspring exposed to simazine identified in the microarray analysis were confirmed by qRT-PCR. The results are reported as the mean ± SEM of six independent testicular analyses performed in triplicate for each group. The data were normalized to the expression level of β-actin and are presented as relative fold changes.

To obtain trancriptomics profiles upon simazine exposure, microarray analysis was performed on RNA isolated from the testes of the 500 µg/kg/day simazine-exposed F1 mice on PND 8 weeks. Applying a twofold threshold, we identified 775 upregulated and 791 downregulated transcripts associated with simazine exposure, among which variable gene categories were affected (Fig. 3A). The array results were validated by performing qRT-PCR on nine different DEGs including Rxfp1 (Fig. 3B).

### Downregulation of Rxfp1 in Simazine-exposed F1 Testes

**Figure 4:**
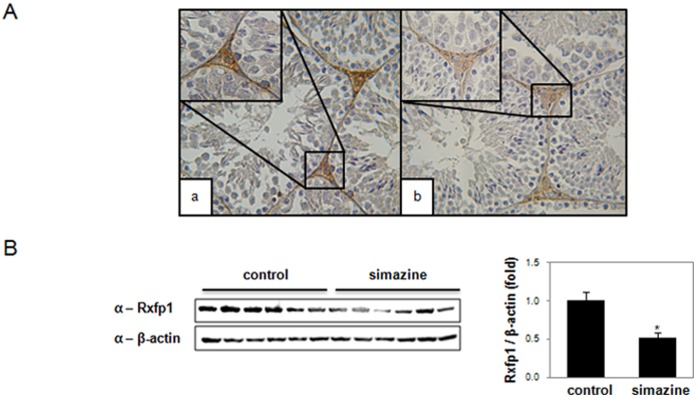
Decreased expression of Rxfp1 in the testis of F1 male mice exposed to simazine. (A) Testicular sections of young adult F1 male control (a) or 500 µg/kg simazine-exposed (b) mice were immunostained for Rxfp1. (B) Six independent testicular lysates of the control and the 500 µg/kg groups were prepared, and western blot analyses were performed using anti-Rxfp1 antibodies (left panel). Equal loading of each sample was confirmed by immunoblotting of the same membrane with anti-β-actin antibody. The results of a quantitative analysis of the Rxfp1 expression shown in the left panel using Multi Gauge V3.0 software are presented in the right panel. The asterisk indicates a significant difference compared with the control (*p*<0.05).

Interestingly, as the mRNA level for Rxfp1, the receptor for a crucial peptide hormone, relaxin [12], was downregulated after simazine exposure (Fig. 3B), an immunohistochemical analysis of Rxfp1 was performed to confirm the observation. In accord, a significant decrease of Rxfp1 protein expression was seen in 500 µg/kg/day simazine-exposed mice testes, and this downregulation was prominent in Leydig cells (Fig. 4A). Furthermore, the level of Rxfp1 protein expressed in the testes of mice exposed to simazine, as determined by western blot analysis, was significantly decreased by more than 0.5-fold (Fig. 4B), showing a consistent and significant downregulation of both the mRNA and the protein of Rxfp1 upon simazine exposure.

### Reduced Expression of Target Genes in the Relaxin-Rxfp1 Pathway in Simazine-exposed F1 Testes

**Figure 5:**
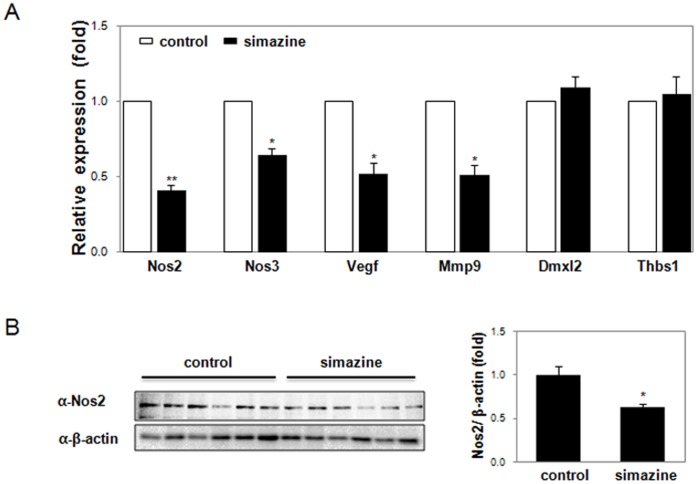
Reduced expression of target genes in the relaxin pathway in the testis of F1 mice exposed to simazine. (A) The expression of crucial target genes (Nos2, Nos3, Vegf, Mmp9) in the relaxin pathway was determined by qRT-PCR on the testis of young adult F1 control or 500 µg/kg simazine-exposed mice. The data (mean ± SEM) are from six testicular analyses performed in triplicate for each group and are shown as relative fold changes. Asterisks indicate significant differences compared with the control (*p<0.05; **p<0.005). (B) The expression of Nos2 protein was further determined by western blot analysis (left panel), and its expression was quantified after normalization to β-actin (n = 6) (right panel). Significantly different values between groups are indicated by an asterisk (*p*<0.05).

To identify downstream target genes in the relaxin-Rxfp1 pathway that may also be affected by simazine-induced Rxfp1 downregulation, the expression of a set of known target genes in the pathway was determined. As shown in Fig. 5A, the mRNA levels of these known target genes, including nitric oxide synthase (Nos) 2 and 3, vascular endothelial growth factor (Vegf), and matrix metallopeptidase 9 (Mmp9), were all significantly decreased in the testes of the 500 µg/kg/day simazine-exposed offspring, whereas the mRNA levels of unrelated genes such as dmX-like protein 2 (Dmxl2) and thrombospondin 1 (Thbs1) were not affected. The downregulation of Nos2, an inducible Nos protein, in simazine-exposed testes was also confirmed by quantitative immunoblot analysis (Fig. 5B).

### Simazine-induced Decreased Rxfp1 Expression and NO Production in Leydig Cells *in vitro*

**Figure 6:**
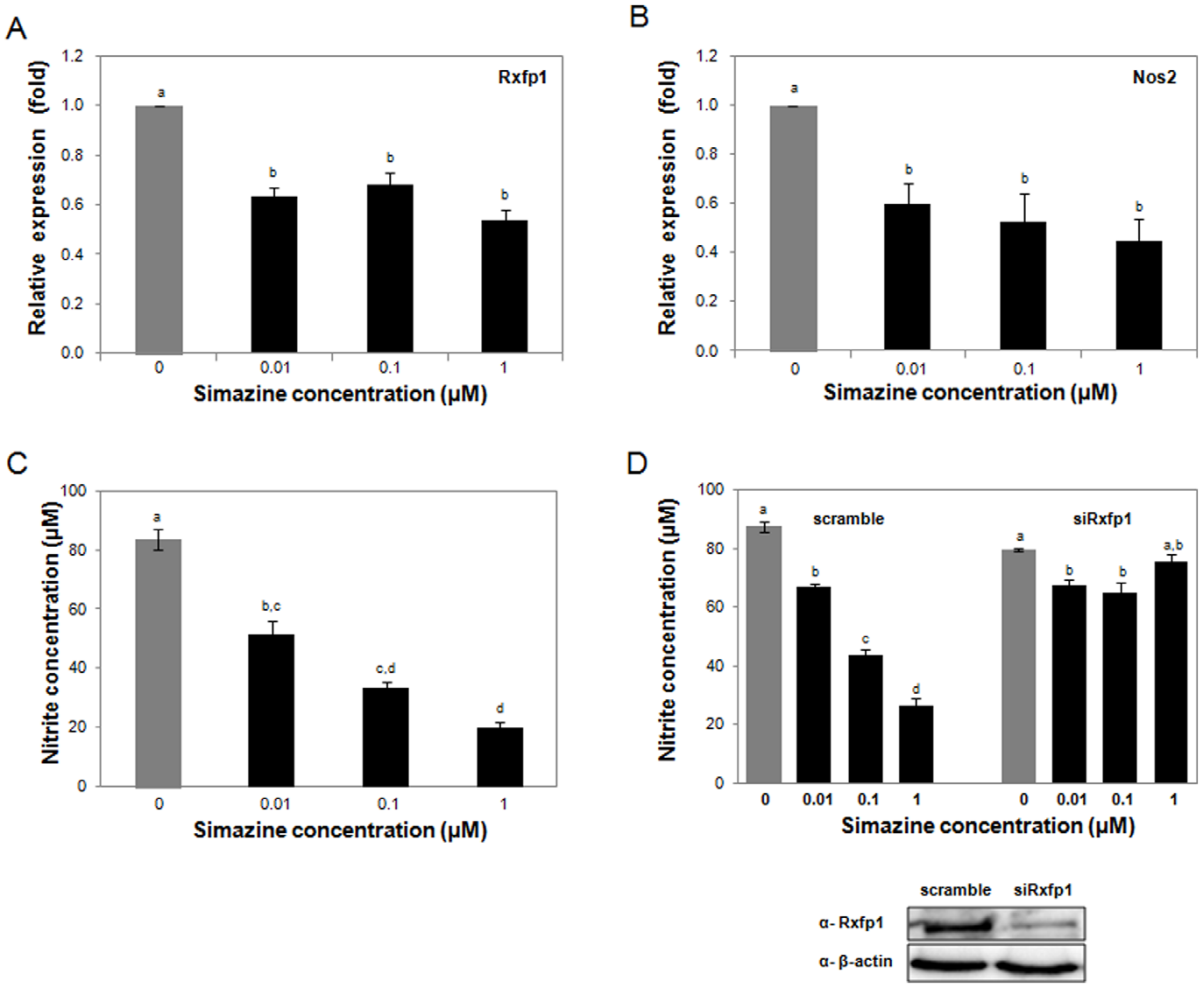
Simazine-induced decreased Rxfp1 and Nos2 expression and reduced NO production in testicular cells *in vitro*. Rat Leydig cells (LC540) were exposed to simazine (0, 0.01, 0.1, or 1 µM) for 36 h, and the mRNA levels of Rxfp1 (A) and Nos2 (B) were analyzed by qRT-PCR. The relative expression levels of Rxfp1 and Nos2 normalized to β-actin are shown. (C) LC540 cells were exposed to various concentrations of simazine for 36 h, and the levels of NO produced were determined by measuring nitrite concentration. (D) LC540 knockdown cells were prepared by transfection with a scrambled sequence or siRNAs for Rxfp1, and reduced Rxfp1 expression was demonstrated by western blotting. Subsequently, knocked-down cells were treated with simazine for 36 h, and the levels of NO produced were determined. For all experiments (A-D), three independent experiments were performed in triplicate, and different letters denote significant values (*p<*0.05).

**Figure 7:**
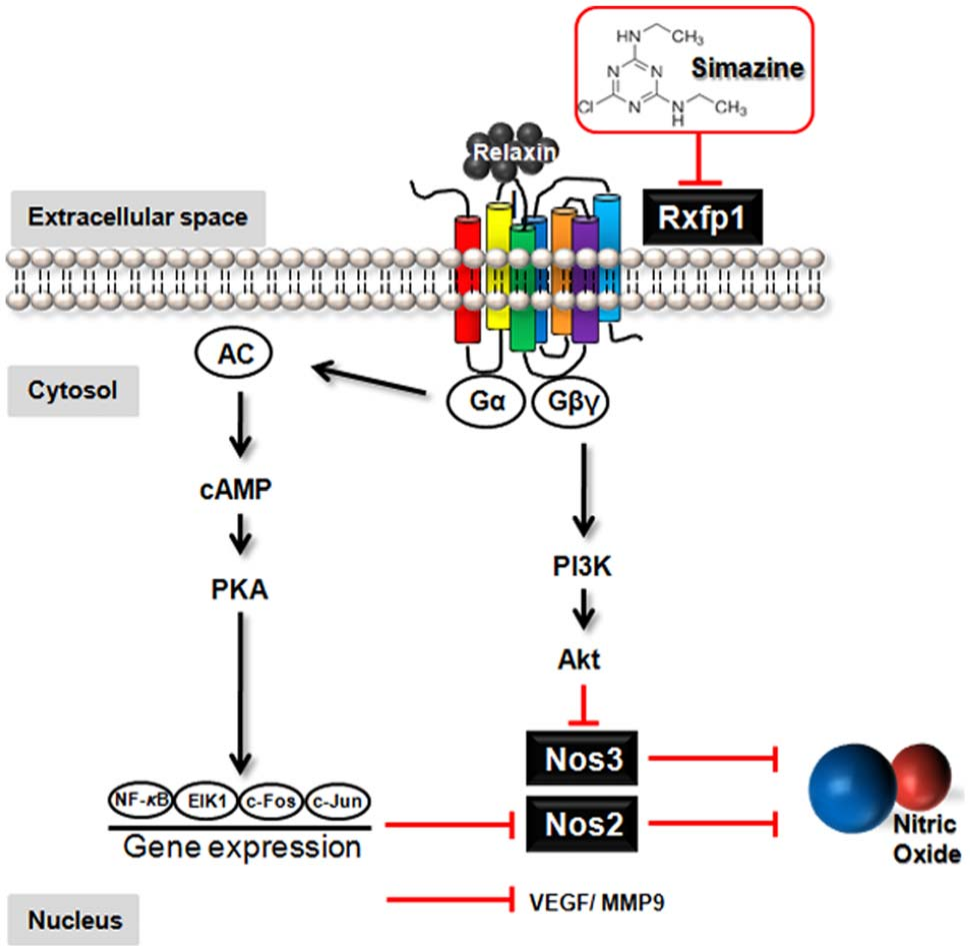
A schematic representation of the relaxin pathway when disturbed by simazine. Simazine inhibits testicular Rxfp1 expression, which consequently may limit the intracellular signaling of relaxin in the testis. The compromised relaxin-Rxfp1 signaling is further expected to downregulate the expression of relaxin target genes such as Nos2, Nos3, Vegf, and Mmp9, leading to the inhibition of NO release.

The binding of relaxin to Rxfp1 increases transcription of its target genes, including Nos2, leading to stimulated production of NO [13], [14]. Thus, to further investigate whether simazine-induced inhibition of the relaxin-Rxfp1 pathway indeed compromises the production of NO, rat Leydig LC450 cells were treated with increasing concentrations (0 to 1 µM) of simazine *in vitro*. Consistent with the *in vivo* mouse data (Fig. 4 and 5), we were also able to observe a significant simazine-induced downregulation of Rxfp1 and Nos2 mRNA in rat Leydig cells, even at a concentration of 0.01 µM (Fig. 6A and B). Prominently, *in vitro* exposure of the Leydig cells to simazine resulted in a significant reduction of NO production in a concentration-dependent manner, even at 0.01 µM (Fig. 6C). Moreover, to determine whether the simazine-induced inhibition of NO release is a consequence of the Rxfp1 downregulation also due to simazine exposure, Rxfp1 expression in the Leydig cells was silenced using a specific siRNA. The knocked-down cells were then exposed to simazine, and NO production was measured. As shown in the Fig. 6D, simazine (0.1 and 1 µM)-mediated inhibition of NO release was significantly diminished in the cells expressing less Rxfp1 protein, suggesting that simazine-induced downregulation of Rxfp1 is likely a mechanism responsible for the inhibited NO production associated with simazine exposure (Fig. 7).

## Discussion

In the present study, we evaluated the effects of simazine exposure on mice during gestation and lactation and found that male offspring that were maternally exposed to simazine during these critical periods showed disturbed growth that manifested in or persisted into adulthood. It is of particular interest that perinatal exposure of simazine at low doses that do not elicit overt maternal toxicity exhibited detrimental effects in offspring that appear later in adult life, supporting the hypothesis that the fetal and neonatal periods are critical for development and that offspring are vulnerable to damage by exposure to simazine during these periods.

Despite its prevalent agricultural use, simazine is considered to have low toxicity, currently available toxicological studies of simazine are limited. To investigate the underlying mechanisms that may contribute to simazine-induced effects on the testis such as enhanced apoptosis and reduced testicular weight and sperm concentration, we performed microarray analyses of neonatal mouse testes to identify changes in the transcriptome profile upon simazine exposure. Rxfp1 was found to be downregulated. Rxfp1, initially known as LGR7, is a G-protein-coupled receptor (GPCR) for a pleiotropic hormone, relaxin [12]. Relaxin was originally classified as a peptide hormone that plays a major role in female reproductive functions during parturition and lactation [15], [16]. Later, the expression of Rxfp1 was also observed in the reproductive tract of the male rat [17]. Although the exact functions of relaxin and Rxfp1 in the testes remain unclear at this point, *Rxfp1*-knockout F_1_ male mice exhibit decreased testicular weight, increased testicular apoptosis, impaired spermatogenesis, and reduced fertility [18]. Strikingly, these phenotypes closely resemble the effects observed in male offspring exposed to simazine, implying that simazine-mediated Rxfp1 downregulation is a likely mechanism responsible for its testicular toxicity. Since simazine exposure decreased both mRNA and protein levels of Rxfp1, simazine likely modulates transcription of Rxfp1 directly or indirectly although the exact mechanism needs to be studied.

The binding of relaxin to Rxfp1 activates G-proteins. The activated G_α_ subunit stimulates adenylyl cyclase (AC) and increases cAMP production, which subsequently activates extracellular signal-regulated kinase (ERK) and NF-_κ_B, leading to stimulation of their target genes, which include Nos2, Vegf, and Mmp9 [13], [19]. Concurrently, the G_βγ_ complex activates the PI3K and Akt pathway, resulting in the phosphorylation-mediated activation of Nos3 [20], [21]. Both pathways are activated upon relaxin binding to Rxfp1, leading to the stimulated production of NO. Three major isoforms of Nos enzymes exist: endothelial Nos (Nos3) and neuronal Nos (Nos1), which are responsible for the continuous basal release of NO, and inducible Nos (Nos2), which is considered to be the more critical isoform, as its expression can be controlled by external factors [22]. NO is a small, diffusible, short-lived free radical gas active in diverse biological responses. In the testis, NO modulates germ cell viability and development and coordinates the testicular production of hormones and cytokines [23]–[26]. It has also been reported that testicular NO production is involved in compromised spermatogenesis in infertile men with varicocele [27]. In this study, simazine-exposed mouse testes showed significant reductions in the expression levels of downstream target genes of the known relaxin-Rxfp1 pathways, especially Nos2 and Nos3 (Fig. 5). In consequence, a decreased level of NO was expected in the testes of simazine-exposed offspring, and correspondingly, we were able to demonstrate simazine-induced reduction of NO production in rat testicular Leydig cells *in vitro* (Fig. 6C). Reduction of lipopolysaccharide-induced NO production by simazine has also been observed in murine macrophage [28].

Apoptosis is a pivotal homeostatic mechanism for maintaining cell populations throughout the development and aging of an organism. Diverse classes of chemicals that elicit toxic responses are mediated by apoptotic cell death. In the present study, we observed increased numbers of apoptotic cells in the testes and the ovaries of mouse offspring that were maternally exposed to simazine (Fig. 2A). To our knowledge, this is the first study to report that simazine can induce apoptosis. The mechanism by which simazine promotes apoptosis is unclear, as the expressions of members of Bcl-2 family, which are pivotal proteins that regulate cellular survival and death, are not prominently altered upon simazine exposure (Fig. 2B). One observation that may explain how simazine promotes apoptosis is the lowered NO production upon simazine exposure (Fig. 6C), which may result in failure to protect cells from apoptosis, since physiological levels of NO are protective [29]–[31].

One of the main characteristics of the simazine-exposed male offspring was smaller body size (Table 3 and 4). Smaller offspring were also reported by other studies after exposure to atrazine [32]–[34], a chemical homologous to simazine, at more than 1000-fold higher doses than the ones we used in the current study. These observations are in accordance with two recent epidemiological studies that identified an association between fetal growth restriction and maternal exposure to atrazine and/or simazine [4], [35], suggesting that maternal exposure to triazine herbicides coherently inhibit normal fetal growth. Although more comprehensive investigational studies are required, the inhibitory activities of simazine toward the relaxin-Rxfp1 signaling pathway demonstrated in this study are a plausible mechanism that can account for the inhibitory effect of simazine on growth. In support of this hypothesis, the anti-proliferative effect of atrazine has been demonstrated in various different cell lines including Chinese hamster ovary cells, human hepatoma cells, and fibroblasts [36]–[38]. It has been shown that relaxin upregulates Nos2 and increases the proliferation of rat Sertoli cells [39]. Moreover, the expression of relaxin is higher in tumors and is strongly correlated with increased proliferation [40]. Downregulation of its receptor, Rxfp1, dramatically reduced tumor growth, as evidenced by decreased proliferation and increased apoptosis of the xenograft tumor [40].

The testis is the central organ in the male reproductive system, being responsible for sperm production. The testis is composed of seminiferous tubules and the interstitial spaces between the tubules. Spermatogenesis is a dynamic and complex process leading to testicular germ cell development [41], [42]. The quality of human semen has been shown to decline over a 50-year period, and epidemiology studies suggest that environmental exposures are associated with the decline in sperm quality [43], [44]. Decreased weights of reproductive and endocrine organs including testis, epididymis, Cowper’s gland, seminal vesicle and thymus gland upon simazine exposure could be a consequence of reduced body weight. However, even though the decrease in organ weight became smaller after adjustment for body weight, the trend was still present (Table 4). The decreased sperm concentrations observed in simazine-exposed mice may be secondary to smaller reproductive organs, especially since sperm quality was not significantly affected (Table 5). At the same time, however, the possibility of a direct effect of simazine on the reproductive function of male mice should be also considered. Careful further investigations are required to explore this association.

The significance of the present study is the identification of relaxin-Rxfp1 signaling as a novel pathway regulated by simazine, which could be associated with the disturbed growth and sperm production elicited upon simazine exposure. In particular, in contrast to previous studies that used doses of hundreds of mg/kg of body weight of atrazine [32]–[34], the present study provides more useful information for risk assessments of simazine not only because the doses of simazine tested in the current study are very low (less than 1000-fold the doses of previous studies) and more similar to those typical of environmental exposure, but also because the maternal simazine exposure at this low doses elicits toxic responses in exposed offspring. Therefore, the results of the current transgenerational animal study suggest that consumption of simazine-contaminated diets by mothers may influence the development of offspring and cause dysfunctional reproductive function. Therefore, a more comprehensive re-evaluation of the risks of triazine-family herbicides is imperative.

## References

[1] Agency EP (2006) Reregistration eligibility decision (RED) document for Simazine List A.

[2] Riviere JE, Brooks JD (2010) Predicting skin permeability from complex chemical mixtures: dependency of quantitative structure permeation relationships on biology of skin model used. Toxicol Sci 119: 224–232. 20947718 10.1093/toxsci/kfq317 PMC3107484

[3] Salasnich L, Sattin F (1995) Charge-exchange processes between excited helium and fully stripped ions. Phys Rev A 51: 4281–4283. 9912107 10.1103/physreva.51.4281

[4] Chevrier C, Limon G, Monfort C, Rouget F, Garlantezec R, et al (2011) Urinary biomarkers of prenatal atrazine exposure and adverse birth outcomes in the PELAGIE birth cohort. Environ Health Perspect 119: 1034–1041. 21367690 10.1289/ehp.1002775 PMC3222984

[5] Belloni V, Dessi-Fulgheri F, Zaccaroni M, Di Consiglio E, De Angelis G, et al (2011) Early exposure to low doses of atrazine affects behavior in juvenile and adult CD1 mice. Toxicology 279: 19–26. 20624442 10.1016/j.tox.2010.07.002

[6] Hovey RC, Coder PS, Wolf JC, Sielken RL Jr, Tisdel MO, et al (2010) Quantitative assessment of mammary gland development in female Long Evans rats following in utero exposure to atrazine. Toxicol Sci 119: 380–390. 21059795 10.1093/toxsci/kfq337

[7] Rohr JR, McCoy KA (2010) A qualitative meta-analysis reveals consistent effects of atrazine on freshwater fish and amphibians. Environ Health Perspect 118: 20–32. 20056568 10.1289/ehp.0901164 PMC2831963

[8] Abarikwu SO, Adesiyan AC, Oyeloja TO, Oyeyemi MO, Farombi EO (2010) Changes in sperm characteristics and induction of oxidative stress in the testis and epididymis of experimental rats by a herbicide, atrazine. Arch Environ Contam Toxicol 58: 874–882. 19672647 10.1007/s00244-009-9371-2

[9] Foradori CD, Hinds LR, Hanneman WH, Handa RJ (2009) Effects of atrazine and its withdrawal on gonadotropin-releasing hormone neuroendocrine function in the adult female Wistar rat. Biol Reprod 81: 1099–1105. 19605789 10.1095/biolreprod.109.077453

[10] Brevini TA, Zanetto SB, Cillo F (2005) Effects of endocrine disruptors on developmental and reproductive functions. Curr Drug Targets Immune Endocr Metabol Disord 5: 1–10. 15777200 10.2174/1568008053174750

[11] Dewson G, Kratina T, Sim HW, Puthalakath H, Adams JM, et al (2008) To trigger apoptosis, Bak exposes its BH3 domain and homodimerizes via BH3:groove interactions. Mol Cell 30: 369–380. 18471982 10.1016/j.molcel.2008.04.005

[12] Hsu SY, Nakabayashi K, Nishi S, Kumagai J, Kudo M, et al (2002) Activation of orphan receptors by the hormone relaxin. Science 295: 671–674. 11809971 10.1126/science.1065654

[13] Bathgate RA, Ivell R, Sanborn BM, Sherwood OD, Summers RJ (2005) Receptors for relaxin family peptides. Ann N Y Acad Sci 1041: 61–76. 15956688 10.1196/annals.1282.010

[14] Masini E, Bani D, Bigazzi M, Mannaioni PF, Bani-Sacchi T (1994) Effects of relaxin on mast cells. In vitro and in vivo studies in rats and guinea pigs. J Clin Invest 94: 1974–1980. 7525651 10.1172/JCI117549 PMC294619

[15] Kohsaka T, Min G, Lukas G, Trupin S, Campbell ET, et al (1998) Identification of specific relaxin-binding cells in the human female. Biol Reprod 59: 991–999. 9746753 10.1095/biolreprod59.4.991

[16] Kuenzi MJ, Sherwood OD (1995) Immunohistochemical localization of specific relaxin-binding cells in the cervix, mammary glands, and nipples of pregnant rats. Endocrinology 136: 1367–1373. 7895647 10.1210/endo.136.4.7895647

[17] Filonzi M, Cardoso LC, Pimenta MT, Queiroz DB, Avellar MC, et al (2007) Relaxin family peptide receptors Rxfp1 and Rxfp2: mapping of the mRNA and protein distribution in the reproductive tract of the male rat. Reprod Biol Endocrinol 5: 29. 17623071 10.1186/1477-7827-5-29 PMC1947996

[18] Krajnc-Franken MA, van Disseldorp AJ, Koenders JE, Mosselman S, van Duin M, et al (2004) Impaired nipple development and parturition in LGR7 knockout mice. Mol Cell Biol 24: 687–696. 14701741 10.1128/MCB.24.2.687-696.2004 PMC343807

[19] Ivell R (2002) Endocrinology. This hormone has been relaxin' too long! Science 295: 637–638. 11809958 10.1126/science.1069234

[20] Nistri S, Bani D (2003) Relaxin receptors and nitric oxide synthases: search for the missing link. Reprod Biol Endocrinol 1: 5. 12646076 10.1186/1477-7827-1-5 PMC151800

[21] Nguyen BT, Yang L, Sanborn BM, Dessauer CW (2003) Phosphoinositide 3-kinase activity is required for biphasic stimulation of cyclic adenosine 3′,5′-monophosphate by relaxin. Mol Endocrinol 17: 1075–1084. 12595573 10.1210/me.2002-0284

[22] Lee NP, Cheng CY (2008) Nitric oxide and cyclic nucleotides: their roles in junction dynamics and spermatogenesis. Adv Exp Med Biol 636: 172–185. 19856168 10.1007/978-0-387-09597-4_10

[23] Weissman BA, Sottas CM, Zhou P, Iadecola C, Hardy MP (2007) Testosterone production in mice lacking inducible nitric oxide synthase expression is sensitive to restraint stress. Am J Physiol Endocrinol Metab 292: E615–620. 17032928 10.1152/ajpendo.00412.2006

[24] Pomerantz DK, Pitelka V (1998) Nitric oxide is a mediator of the inhibitory effect of activated macrophages on production of androgen by the Leydig cell of the mouse. Endocrinology 139: 922–931. 9492021 10.1210/endo.139.3.5773

[25] O'Bryan MK, Schlatt S, Gerdprasert O, Phillips DJ, de Kretser DM, et al (2000) Inducible nitric oxide synthase in the rat testis: evidence for potential roles in both normal function and inflammation-mediated infertility. Biol Reprod 63: 1285–1293. 11058531 10.1095/biolreprod63.5.1285

[26] Andric SA, Janjic MM, Stojkov NJ, Kostic TS (2010) Testosterone-induced modulation of nitric oxide-cGMP signaling pathway and androgenesis in the rat Leydig cells. Biol Reprod 83: 434–442. 20463352 10.1095/biolreprod.110.083626

[27] Shiraishi K, Naito K (2007) Nitric oxide produced in the testis is involved in dilatation of the internal spermatic vein that compromises spermatogenesis in infertile men with varicocele. BJU Int 99: 1086–1090. 17346270 10.1111/j.1464-410X.2007.06800.x

[28] Kim KR, Son EW, Rhee DK, Pyo S (2002) The immunomodulatory effects of the herbicide simazine on murine macrophage functions in vitro. Toxicol In Vitro 16: 517–523. 12206818 10.1016/s0887-2333(02)00047-4

[29] Marathe N, Rangaswami H, Zhuang S, Boss GR, Pilz RB (2011) Pro-survival effects of 17beta-estradiol on osteocytes are mediated by nitric oxide/cGMP via differential actions of cGMP-dependent protein kinases I and II. J Biol Chem 287: 978–988. 22117068 10.1074/jbc.M111.294959 PMC3256896

[30] Zamberlam G, Portela V, de Oliveira JF, Goncalves PB, Price CA (2011) Regulation of inducible nitric oxide synthase expression in bovine ovarian granulosa cells. Mol Cell Endocrinol 335: 189–194. 21256181 10.1016/j.mce.2011.01.013

[31] Jee BC, Kim SH, Moon SY (2003) The role of nitric oxide on apoptosis in human luteinized granulosa cells. Immunocytochemical evidence. Gynecol Obstet Invest 56: 143–147. 14530614 10.1159/000073773

[32] Fraites MJ, Narotsky MG, Best DS, Stoker TE, Davis LK, et al (2011) Gestational atrazine exposure: effects on male reproductive development and metabolite distribution in the dam, fetus, and neonate. Reprod Toxicol 32: 52–63. 21530639 10.1016/j.reprotox.2011.04.003

[33] Victor-Costa AB, Bandeira SM, Oliveira AG, Mahecha GA, Oliveira CA (2010) Changes in testicular morphology and steroidogenesis in adult rats exposed to Atrazine. Reprod Toxicol 29: 323–331. 20045047 10.1016/j.reprotox.2009.12.006

[34] Rayner JL, Enoch RR, Wolf DC, Fenton SE (2007) Atrazine-induced reproductive tract alterations after transplacental and/or lactational exposure in male Long-Evans rats. Toxicol Appl Pharmacol 218: 238–248. 17204298 10.1016/j.taap.2006.11.020

[35] Ochoa-Acuna H, Frankenberger J, Hahn L, Carbajo C (2009) Drinking-water herbicide exposure in Indiana and prevalence of small-for-gestational-age and preterm delivery. Environ Health Perspect 117: 1619–1624. 20019915 10.1289/ehp.0900784 PMC2790519

[36] Powell ER, Faldladdin N, Rand AD, Pelzer D, Schrunk EM, et al (2011) Atrazine exposure leads to altered growth of HepG2 cells. Toxicol In Vitro 25: 644–651. 21232595 10.1016/j.tiv.2011.01.001

[37] Kmetic I, Gaurina Srcek V, Slivac I, Simic B, Kniewald Z, et al (2008) Atrazine exposure decreases cell proliferation in Chinese Hamster Ovary (CHO-K1) cell line. Bull Environ Contam Toxicol 81: 205–209. 18465069 10.1007/s00128-008-9425-6

[38] Manske MK, Beltz LA, Dhanwada KR (2004) Low-level atrazine exposure decreases cell proliferation in human fibroblasts. Arch Environ Contam Toxicol 46: 438–444. 15253040 10.1007/s00244-003-3087-5

[39] Cardoso LC, Nascimento AR, Royer C, Porto CS, Lazari MF (2010) Locally produced relaxin may affect testis and vas deferens function in rats. Reproduction 139: 185–196. 19812235 10.1530/REP-09-0146

[40] Feng S, Agoulnik IU, Bogatcheva NV, Kamat AA, Kwabi-Addo B, et al (2007) Relaxin promotes prostate cancer progression. Clin Cancer Res 13: 1695–1702. 17363522 10.1158/1078-0432.CCR-06-2492

[41] Carreau S, Bilinska B, Levallet J (1998) [Male germ cells. A new source of estrogens in the mammalian testis]. Ann Endocrinol (Paris) 59: 79–92. 9789591

[42] O'Donnell L, Robertson KM, Jones ME, Simpson ER (2001) Estrogen and spermatogenesis. Endocr Rev 22: 289–318. 11399746 10.1210/edrv.22.3.0431

[43] Swan SH (2006) Semen quality in fertile US men in relation to geographical area and pesticide exposure. Int J Androl 29: 62–68; discussion 105–108. 10.1111/j.1365-2605.2005.00620.x 16466525

[44] Jurewicz J, Hanke W, Radwan M, Bonde JP (2009) Environmental factors and semen quality. Int J Occup Med Environ Health 22: 305–329. 20053623 10.2478/v10001-009-0036-1

